# Bio-Based Nanocellulose Cryogels Modified with Tannin and Vanillin: Intermolecular Interactions and Functional Properties

**DOI:** 10.3390/polym18121529

**Published:** 2026-06-19

**Authors:** Lincoln Audrew Cordeiro, Alessandro Zanchin, Elena Colusso, Camila Monteiro Cholant, Patricia Oliveira Schmitt, Radmila Rodrigues Gravato, Lorenzo Moro, Mara Vegro, Sarah Kalli Silva da Silva, Amanda Marcely Reis, Jonas Raphael Eckardt, Lorenzo Guerrini, André Luiz Missio, Gianluca Tondi

**Affiliations:** 1 Graduate Program in Science and Materials Engineering, Technological Development Center—CDTec, Federal University of Pelotas (UFPel), Gomes Carneiro, 1, Pelotas 96010-610, RS, Brazil; lincoln.cordeiro@ufpel.edu.br (L.A.C.); andre.missio@ufpel.edu.br (A.L.M.); 2 TESAF Department, University of Padua, Viale dell’Università 16, 35020 Legnaro, Italy; 3 Department of Industrial Engineering, University of Padua, Via Marzolo 9, 35131 Padua, Italy; elena.colusso@unipd.it (E.C.); lorenzo.moro@unipd.it (L.M.); 4 Graduate Program in Environmental Sciences, Center for Engineering, Benjamin Constant, 989, Pelotas 96010-020, RS, Brazil; 5 DAFNAE Department, University of Padua, Viale dell’Università 16, 35020 Legnaro, Italy; 6 Graduate in Materials Engineering, Technological Development Center—CDTec, Federal University of Pelotas (UFPel), Gomes Carneiro, 1, Pelotas 96010-610, RS, Brazil

**Keywords:** renewable architectures, supramolecular organization, porosity, lignocellulosic networks, lightweight monoliths

## Abstract

Sustainable lightweight materials based on renewable resources have attracted increasing attention as alternatives to synthetic materials. However, developing nanocellulose cryogels with adequate structural integrity and efficient retention of phenolic compounds remains challenging, often requiring furanic and dialdehyde-based additives associated with environmental and health concerns. In this context, tannin-containing nanocellulose cryogels were produced using vanillin and hydrogen peroxide as sustainable modification agents. The effects of the additives on the structural, morphological, colorimetric, mechanical, thermal, and leaching properties of the cryogels were investigated. FTIR and colorimetric analyses revealed the presence of phenolics and the effect of hydrogen peroxide. SEM analysis showed that tannin promoted structural densification, whereas peroxide induced fragmentation of the cryogel network and pore reorganization. These changes influenced density and mechanical performance, with nanocellulose-tannin exhibiting the highest compressive strength and elastic modulus. Thermal conductivity values remained within the range reported for highly porous lignocellulosic materials (38.93–43.79 (mW/m·K)). Tannin leaching demonstrated that peroxide significantly improved tannin retention, especially in the system including vanillin which exhibited only 13,61% tannin release. Overall, vanillin and hydrogen peroxide modified the supramolecular organization and functional properties of the cryogels, highlighting their potential as additives in porous materials for thermal insulation and adsorption applications.

## 1. Introduction

The development of high-performance materials with insulating properties has been increasingly driven by the search for sustainable alternatives based on renewable biomass, aiming to replace fossil-based materials [1,2,3]. In this context, cryogels have emerged as promising materials due to their high porosity, low density, and large specific surface area, being composed predominantly of air (up to ~95%) [4,5]. These characteristics enable their application in thermal and acoustic insulation, biosorbents for aqueous and gaseous contaminants, as well as gas sensing devices [1,6,7,8].

Among the different systems investigated, nanocellulose-based cryogels have attracted significant attention, as they combine the intrinsic advantages of highly porous structures with the unique properties of nanocellulose, including abundance, renewability, biocompatibility, low toxicity, high mechanical strength, elevated Young’s modulus, and ease of surface chemical modification [6,8,9]. The freeze-drying process promotes the formation of an interconnected three-dimensional network of nanofibrils, resulting in lightweight, porous, and structurally stable materials [10,11,12].

Due to the high amount of hydroxyl groups present in nanocellulose, the incorporation of naturally derived phenolic compounds has been explored as a promising strategy for the chemical modification and functionalization of polysaccharide matrices [8,13]. These compounds exhibit higher chemical reactivity and the ability to establish intermolecular interactions with cellulose, contributing both to structural reinforcement and to the introduction of chemically active sites within the network [14,15,16]. In this regard, tannins and vanillin stand out as particularly relevant additives for the development of functional cryogels [17,18].

Tannins are naturally occurring polyphenolic macromolecules widely distributed in various parts of plants. Their aromatic structure, rich in phenolic groups, provides high chemical reactivity and enables interactions with polysaccharides through primary interactions (in the presence of modifying agents) and secondary interactions, such as hydrogen bonding, π–π interactions, and other van der Waals forces [19,20,21,22,23,24]. In addition, tannins exhibit a strong ability to chelate metal ions, as deprotonated phenolic groups can coordinate with metal species such as Fe^2+^ and Cu^2+^ in aqueous systems, making them attractive for wastewater treatment applications [25,26,27]. However, due to their solubility and relatively weak direct interaction with nanocellulose, the use of modifying agents is often required to enhance their incorporation and improve structural stability within the matrix [25,28,29,30]. In addition to their natural abundance and renewability, tannins are particularly attractive for cryogel functionalization due to their multifunctional aromatic structure, which may contribute not only to intermolecular stabilization but also to adsorption-related properties through phenolic active sites [25].

Conventional crosslinking agents, including glutaraldehyde, glyoxal, formaldehyde, and furfuryl alcohol-based systems, have been widely employed to improve the structural stability of tannin-based materials with or without lignocellulosic biomass. These compounds promote the formation of three-dimensional networks through covalent bonding reactions involving hydroxyl and phenolic groups, resulting in enhanced rigidity, dimensional stability, and mechanical performance [31,32,33,34,35,36]. However, concerns regarding the toxicity of these compounds and their potential environmental and health impacts have encouraged the search for more sustainable alternatives. To address this challenge, naturally derived compounds such as vanillin and hydrogen peroxide have attracted increasing interest as a modification strategy [37]. Nevertheless, their combined use for the development of nanocellulose–tannin cryogels has not yet been investigated.

Vanillin, a phenolic aldehyde naturally found in vanilla pods, is widely used as a flavoring agent, fragrance component, and intermediate in cosmetic and pharmaceutical industries [38]. In the literature, most studies involving vanillin as a modifying agent are focused on chitosan-based systems, where typically interactions occur via Schiff base formation and intermolecular hydrogen bonding. These systems have been explored for applications in packaging [39,40], biomedical materials [41], and adsorbents [42]. However, the interaction mechanism in cellulose-rich matrices is expected to differ due to the predominance of hydroxyl groups rather than amino functionalities. In cellulose-based systems, intermolecular organization is largely governed by extensive interactions with hydrogen bonding between the phenolic hydroxyl group of vanillin and the hydroxyl/carboxyl groups of cellulose (as demonstrated in carboxymethyl cellulose and hydroxyethyl cellulose systems), as well as other minor dipolar secondary forces; in rare situations, also hemiacetal-type associations via reversible interaction between the aldehyde group of vanillin and the hydroxyl groups of cellulose can occur [43,44,45]. Therefore, the application of vanillin in nanocellulose–tannin systems represents a promising approach for the development of sustainable, functional cryogels with enhanced structural and performance properties. Alternatively, oxidative processes can affect fiber–fiber and fiber–phenolic compound interactions by promoting the formation of new reactive sites and oxidative coupling reactions, thereby enhancing network cohesion, structural stability, and the mechanical performance of nanocellulose-based cryogels [46,47]. Despite these advances, systematic comparative studies addressing the effects of different modification mechanisms on mechanical performance, chemical interactions, and tannin leaching in nanocellulose cryogels remain limited.

This study reports the development of nanocellulose-based cryogels functionalized with tannin and vanillin and hydrogen peroxide as possible hardeners. The cryogels were characterized for their structural, chemical, morphological, thermal, and mechanical properties, as well as their tannin leaching behavior. By integrating these different characterization techniques, the work aims to better understand how the presence of vanillin and hydrogen peroxide affects the chemistry and the morphology of tannin–nanocellulose network, supporting the advancement of sustainable porous materials from renewable lignocellulosic resources.

## 2. Materials and Methods

### 2.1. Materials

Bleached *Eucalyptus* cellulose, with a moisture content of 65.92%, was obtained from the Federal University of Santa Maria (UFSM) and used as the matrix for cryogel preparation. Condensed tannin from mimosa (*Acacia mearnsii*), commercially named Seta RC and supplied by Seta S.A., with a moisture content of 7%, was used as the immobilized additive. Vanillin (PA) and hydrogen peroxide (30%*v*/*v*, P.A.–ACS grade), both from Dinâmica Química Contemporânea, were employed as possible hardeners.

### 2.2. Preparation of Nanocellulose-Based Hybrid Suspensions

**Figure 1:**
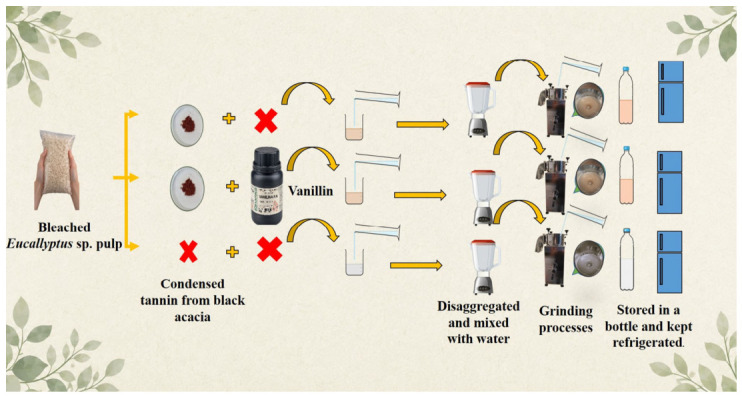
Production scheme for suspension processes.

The preparation of the nanocellulose suspensions was based on the methodology reported by Coldebella et al. [11] and is illustrated in Figure 1 and Table 1. Suspensions with a solid concentration of 2 wt% (20 g of dry material) were initially produced. The formulations containing cellulose, tannin, and vanillin in mass proportions of 100:0:0, 96:4:0, and 95:4:1 (wt%), respectively, were first dispersed in 1000 mL of distilled water using a laboratory blender to ensure uniform mixing. Subsequently, another 1000 mL of distilled water was incorporated, and the mixtures were mechanically processed in a Super Masscolloider Masuko mill (Project Inox, Masuko Sangyo Co., Ltd., Kawaguchi, Japan) at 1700 rpm for 15 cycles. The resulting suspensions were then maintained at 4 °C under refrigeration until further application in the cryogel preparation stage.

### 2.3. Cryogel Production

**Figure 2:**
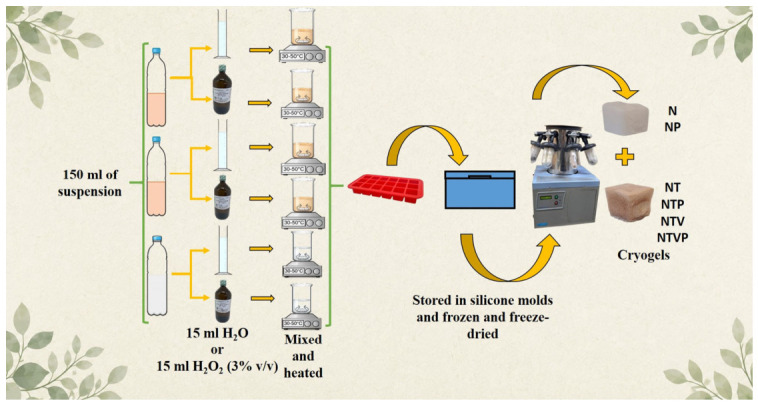
Production scheme for cryogel processes.

Approximately 150 mL of each prepared suspension was collected and mixed with either 15 mL of distilled water or 15 mL of a 3% (*w*/*v*) hydrogen peroxide solution under magnetic stirring at temperatures ranging from 30 to 50 °C for 30 min. Preliminary trials were conducted using 2, 4, 6, and 8 wt% tannin. Increasing the tannin content above 4 wt% resulted in higher density, irregular tannin distribution throughout the cryogel structure, and, at 8 wt%, visible phase separation took place. Therefore, 4 wt% was selected as the highest tannin concentration suitable for producing cryogels under the conditions employed in this study. The selected suspensions were poured into silicone molds with dimensions of 3 × 3 × 2 cm^3^, yielding six cryogels for each formulation. Subsequently, the samples were stored in a freezer at −17 °C for 24 h and then freeze-dried using a Liobras freeze dryer, Liobras, São Carlos, Brazil (Liotop model L202, 250 mmHg, −35 °C) for 72 h (Figure 2).

### 2.4. Characterization

#### 2.4.1. Fourier Transform Infrared Spectroscopy (FTIR)

Fourier transform infrared spectroscopy (FTIR) was employed to identify the functional groups present and to investigate the chemical interactions between nanocellulose, tannin, and the modifying agents. FTIR spectra were recorded using a Thermo Nicolet spectrometer equipped with an attenuated total reflectance (ATR) accessory (Thermo Fisher Scientific, Waltham, MA, USA), operating with a resolution of 4 cm^−1^ and 16 scans, over the spectral range from 4000 to 400 cm^−1^.

#### 2.4.2. Colorimetric Analysis

The colorimetric properties of the cryogels were evaluated using a portable digital colorimeter (Eoptis, model CLM-194, Trento, Italy), equipped with color analysis and classification software. The cryogel samples were firstly compressed and successively tested, measuring the color according to the (CIE Lab*) color space system and obtaining the parameters L* (lightness), a* (red–green coordinate), and b* (yellow–blue coordinate).

#### 2.4.3. Scanning Electron Microscopy (SEM)

Scanning electron microscopy (SEM) analyses were performed using a FEI Quanta 200 microscope (FEI, Eindhoven, The Netherlands), operated at an accelerating voltage of 20 kV under low-vacuum conditions. Micrographs were obtained at magnifications of 50× and 1500×, corresponding to scales of 2 mm and 50 µm.

#### 2.4.4. Bulk Density

Images were acquired using a Canon R10 RGB camera (Canon Inc., Tokyo, Japan; 24.2 MP) equipped with a Canon RF-S 18–150 mm lens, operating in aperture priority mode (f/3.5) with manual focus [48]. The camera rotated around the samples at a fixed distance of approximately 450 mm, capturing 33 images per revolution at three vertical angles (0°, +45°, and −45°) under controlled LED illumination. A reference cube with fiducial markers was used for dimensional calibration. Three-dimensional (3D) reconstruction and image processing were performed using Metashape Professional 2.0.3 (Agisoft LLC, St. Petersburg, Russia). The accuracy of the reconstructed models was verified based on marker alignment and cube dimensions. The external volume obtained from the 3D models was used to calculate the apparent (bulk) density of the samples (kg/m^3^), providing a more representative assessment of the physical properties of the cryogels.

#### 2.4.5. Mechanical Properties

Mechanical compression tests were carried out on ten cryogel specimens from each formulation (dimensions: 3 × 3× 2.5 cm^3^) to determine the maximum compressive force (F_max_), elastic modulus (MOE), and the percentage retention of maximum force (F_max2_/F_max1_ (%)). The tests were performed using a Galdabini Quasar 25 universal testing machine equipped with a 100 N load capacity (Galdabini, Cardano al Campo, Italy), following an adapted version of ISO 844:2021 [49].

The F_max_ and MOE were determined from the first compression cycle, representing the load-bearing capacity and the resistance to elastic deformation of the cryogel structure under compressive stress. The (F_max2_/F_max1_ (%)), calculated as the ratio between the F_max_ values obtained in the first and second compression cycles, was used to investigate the degree of mechanical recovery and structural reversibility of the cryogels.

#### 2.4.6. Thermal Conductivity

Thermal conductivity was determined using the transient plane source (TPS) method with a Hot Disk TPS 3500 apparatus (Hot Disk AB, Gothenburg, Sweden) [50,51], using the software module dedicated to high insulation materials. A double-spiral nickel foil sensor insulated with Kapton layers was sandwiched between two cryogel samples of identical composition, each measuring 4.5 × 4.5 × 2.5 cm^3^. The sensor acted simultaneously as a planar heat source and as a resistance thermometer, providing the heat input and recording the temperature rise on the active sample surfaces as a function of time. A Kapton sensor 8563 (radius: 9.863 mm) was chosen, with an applied power of 10 mW and a measurement time of 40 s. For each formulation, one sample pair was measured five consecutive times, and the average value was reported.

#### 2.4.7. Tannin Leaching

To evaluate the degree of tannin interaction within the nanocellulose matrix, a method adapted from Cordeiro et al. [25] was employed. 0.01 g of each cryogel formulation was collected and individually dispersed in 10 mL of distilled water in Falcon tubes for 30 min without further agitation. Subsequently, the samples were centrifuged at 1000 rpm for 5 min, and the supernatants were collected and filtered using sterile polyethersulfone (PES) syringe filters with a pore size of 0.45 µm. The filtered solutions were analyzed using a Varian Cary 50 Bio UV–Vis spectrophotometer (Varian Inc., Mulgrave, Australia), and absorbance values were recorded at a wavelength of 280 nm.

The amount of tannin leached from the cryogel samples was estimated using the Beer–Lambert law, where *x* represents the concentration of leached phenolics (mg/mL), and *y* corresponds to the measured absorbance. The calibration curve was constructed using tannin standard solutions with 8 concentrations ranging from 0.0312 to 0.003906 mg/mL (T, TP, TV, TVP, respectively). The results of the reading were expressed in concentration (mg/mL) and also as a relative concentration in % according to the following formula:

$$Relative\;concentration\left(\%\right)=\;\frac{concentration\;of\;phenolics\;in\;the\;leached\;sample}{theoretic\;maximum\;concentration\;of\;phenolics\;from\;the\;cryogel\;}\;\times 100$$

#### 2.4.8. Statistical Analysis

All experiments were performed in replicates, and the results are presented as mean ± standard deviation. Prior to statistical comparisons, the homogeneity of variances was evaluated using Levene’s and Bartlett’s tests. Subsequently, the data were analyzed by one-way analysis of variance (ANOVA), followed by Tukey’s post hoc test for multiple comparisons at a significance level of 1% (*p* < 0.01).

## 3. Results

### 3.1. FTIR

**Figure 3:**
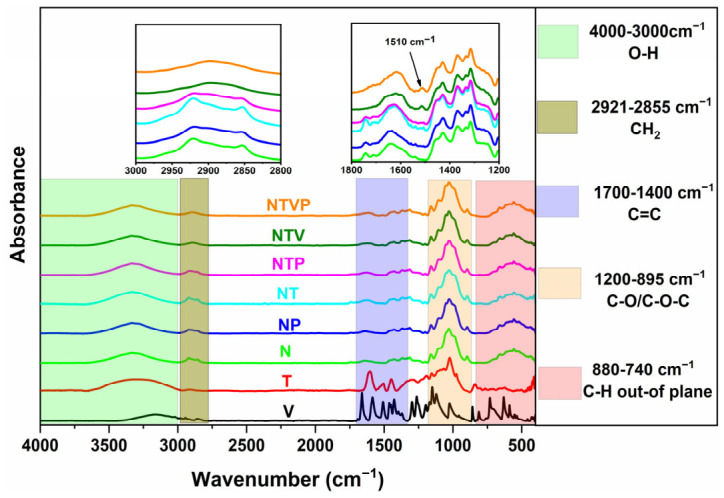
FT-IR spectra of Mimosa tannin and the cryogels object of this study.

The FTIR spectra of cryogels and precursors are presented below to highlight the differences occurring among the different treatments (Figure 3).

The spectral region between 4000 and 3000 cm^−1^ corresponds predominantly to O–H stretching vibrations, reflecting the presence of hydroxyl groups from both cellulose and tannin structures. In the range of 1700–1400 cm^−1^, the absorption bands are mainly related to aromatic C=C stretching vibrations associated with phenolic constituents. The fingerprint region (1200–895 cm^−1^) is characterized by C–O and C–O–C stretching vibrations, which are attributed to tannin functional groups and to the glycopyranosidic backbone of cellulose. Additionally, bands observed between 880 and 740 cm^−1^ are associated with tannin structures, particularly due to out-of-plane deformations of aromatic rings [52,53,54]. Overall, the spectra of nanocellulose and nanocellulose/tannin systems showed very similar vibrational profiles, both in the absence and presence of hydrogen peroxide, indicating that tannin incorporation does not significantly affect the chemical structure of the matrix. This behavior is attributed to the predominance of nanocellulose, whose characteristic bands dominate the spectra and overlap with tannin contributions.

After the addition of hydrogen peroxide, a decrease in the intensity of the bands at ~2921 and ~2855 cm^−1^ was observed, corresponding to the asymmetric and symmetric stretching vibrations of CH_2_ groups. This spectral change suggests alterations in the vibrational environment associated with aliphatic groups after peroxide treatment. Since a similar trend was observed in both tannin-free and tannin-containing samples, the effect appears to be mainly related to the presence of hydrogen peroxide. In contrast, the incorporation of vanillin led to more evident spectral changes. In samples containing 1% vanillin, the bands at ~2921 and ~2855 cm^−1^ became less resolved and merged into a broader band centered around ~2900 cm^−1^, indicating changes in the vibrational environment of CH_2_ groups. In IR spectroscopy, the observation of smoother bands is often due to the increase in complexity and in particular in this case we attribute it to an increase in interaction between components.

Additionally, a band at ~1510 cm^−1^, attributed to aromatic ring vibrations, became visible in vanillin-containing samples. However, considering the relatively low concentration of vanillin and the detection limits of FTIR, this band cannot be exclusively attributed to vanillin and may also be associated with the increased contribution of aromatic phenolic structures within the system. The similarity between the spectra of vanillin-containing samples, in the presence and absence of hydrogen peroxide, indicates that peroxide treatment had a limited effect on the overall vibrational profile of the system.

### 3.2. Colorimetric Analysis

**Figure 4:**
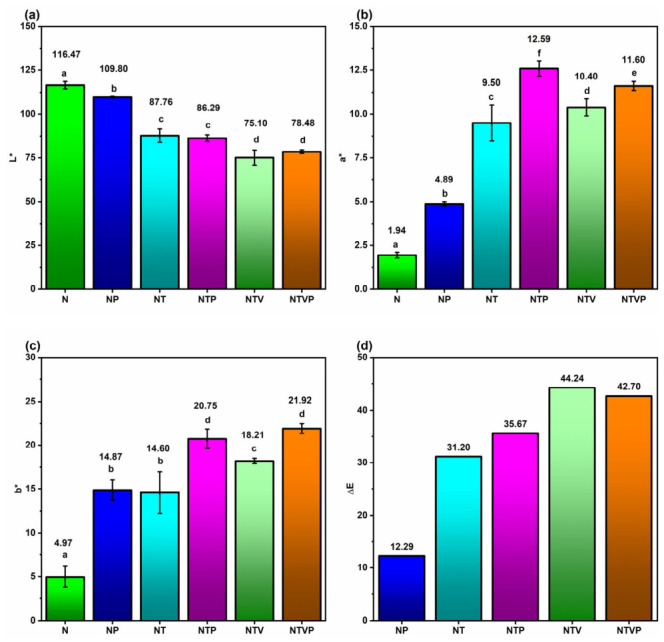
Colorimetric analysis of the cryogel samples investigated in this study: (**a**) lightness (L*); (**b**) red–green coordinate (a*); (**c**) yellow–blue coordinate (b*); and (**d**) total color difference (ΔE). N, NP, NT, NTP, NTV, and NTVP correspond to the cryogel formulations described in Table 1. Different letters within the same graphic indicate statistically significant differences according to Tukey’s test at the 1% significance level (*p* < 0.01).

Colorimetric analysis was performed to evaluate the effect of tannin, vanillin, and hydrogen peroxide incorporation on the optical properties of the cryogels. The parameters L*, a*, b*, and total color difference (ΔE) were used to investigate variations in luminosity and chromatic behavior associated with the presence of aromatic structures, oxidative processes, and intermolecular interactions within the cryogel network (Figure 4).

For L*, the samples showed a progressive reduction in luminosity with the incorporation of tannin, vanillin, and hydrogen peroxide (Figure 4a). N presented the highest L* value (116.47), followed by NP (109.80), indicating that hydrogen peroxide alone caused only a slight reduction in brightness. This decrease may be attributed to the formation of carbonyl groups, including aldehydes and ketones, generated during partial cellulose oxidation. These compounds can act as precursors of chromophoric structures, contributing to yellowing and consequently reducing the L* value [55]. The incorporation of tannin significantly reduced the L* values in NT (87.76) and NTP (86.29), while vanillin-containing systems exhibited the most pronounced darkening, especially NTV (75.10) and NTVP (78.48). This behavior is consistent with the FTIR analysis, which revealed the appearance and intensification of the aromatic band at ~1510 cm^−1^ in vanillin-containing samples, associated with conjugated aromatic structures capable of absorbing visible light and reducing luminosity [56].

For a* (Figure 4b), all modified systems exhibited higher values than the control sample N (1.94), indicating an increase in the red component of the materials. The highest value was observed for NTP (12.59), followed by NTVP (11.60) and NTV (10.40). The increase in a* values is associated with the structural modifications observed by FTIR, particularly the changes in the ~2900 cm^−1^ and 1510 cm^−1^ region and the increased contribution of aromatic vibrations, suggesting enhanced intermolecular interactions and formation of chromophoric structures within the matrix. In addition, phenolic compounds generally exhibit a natural tendency toward reddish coloration, which further supports the observed behavior [57].

Similarly, b* values (Figure 4c) progressively increased with the incorporation of additives, indicating intensification of the yellow component. N showed the lowest value (4.97), while NTP (20.75) and NTVP (21.92) presented the highest values. The higher b* values in peroxide-containing systems are in agreement with the FTIR results, which demonstrated a reduction in the intensity of the CH_2_ stretching bands at ~2921 and ~2855 cm^−1^ after hydrogen peroxide incorporation. These spectral changes suggest oxidative modifications in the aliphatic environment, favoring the formation of conjugated chromophoric structures associated with yellow coloration [58].

The total color difference (ΔE) also progressively increased (Figure 4d), following the order NP (12.29) < NT (31.20) < NTP (35.67) < NTVP (42.70) < NTV (44.24). This behavior confirms that the incorporation of tannin, hydrogen peroxide, and especially vanillin promoted cumulative structural modifications that directly affected the optical properties of the cryogels. Overall, the correlation between FTIR and colorimetric analyses demonstrates that the molecular and structural reorganizations induced by the additives were directly reflected in the visual appearance of the materials.

### 3.3. SEM

**Figure 5:**
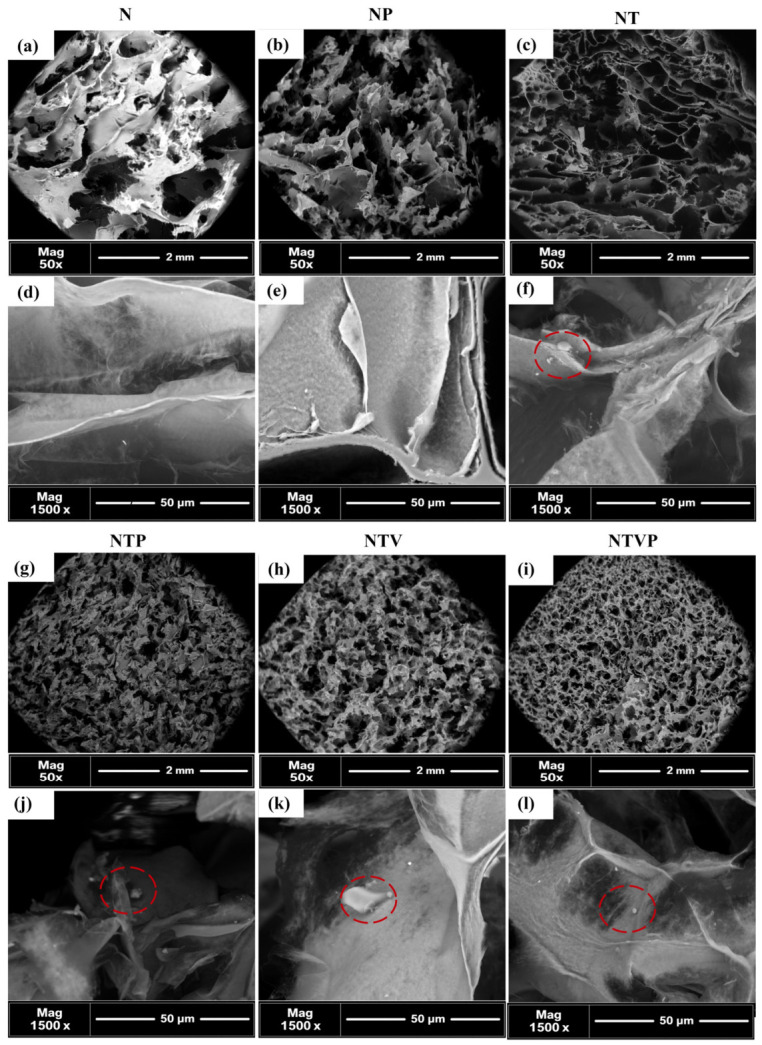
SEM images of cryogels with magnifications of 50× (2 mm) and 1500× (50 µm): (**a**,**d**) N; (**b**,**e**) NP; (**c**,**f**) NT; (**g**,**j**) NTP; (**h**,**k**) NTV; and (**i**,**l**) NTVP. Images (**a**–**c**,**g**–**i**) correspond to 50× magnification, whereas images (**d**–**f**,**j**–**l**) correspond to 1500× magnification. Red dashed circles indicate granular features observed on the cryogel.

The micrographs of the materials are presented in Figure 5 in backscattered electrons (BSD) in order to investigate the morphological interactions between the additives and the nanocellulosic matrix. To complement the qualitative observations, a quantitative pore image analysis was performed on SEM micrographs acquired at the 2 mm scale. The results are summarized in Table 2.

The analysis of pure nanocellulose (N) revealed the formation of well-defined lamellar structures characterized by relatively large void domains and a low number of detectable pores (0.013 mm^2^, n/mm^2^ = 119.75), which are typical features resulting from the freezing process and the directional growth of ice crystals. Upon the addition of hydrogen peroxide (NP), the structure became more fragmented, exhibiting smaller and less continuous lamellae, as well as thinner and more fibrillated extremities. These morphological changes suggest partial disruption of the nanocellulose network. Quantitative image analysis supported these observations, revealing a redistribution of the porous architecture toward a greater number of smaller void domains.

The incorporation of tannin (NT) promoted fiber aggregation, reducing the spacing between adjacent layers and generating a more locally compact structure. Compared with N, NT exhibited a higher number of pores (n/mm^2^ = 202.5) and a lower average void area (0.005 mm^2^), indicating that tannin-induced interactions contributed to the formation of a denser architecture composed of smaller cavities. The introduction of hydrogen peroxide into this system (NTP) partially mitigated the aggregation effect, resulting in a more fragmented morphology with fewer effective interaction sites available for lamellar assembly. Consequently, NTP presented the highest pore count and the lowest average void area among all formulations, suggesting that hydrogen peroxide promoted the subdivision of larger cavities into a greater number of smaller void domains. This behavior is consistent with the FTIR results, which indicated that the oxidative treatment affected the aliphatic segments of the nanocellulosic matrix, potentially influencing fibril organization and pore development during cryogel formation.

For the NTV sample, a predominantly fragmented morphology was observed, characterized by a less cohesive three-dimensional network and more open, interconnected pores. This structure indicates a lower degree of densification and a larger volume of accessible void space, which is supported by the high total void area (7.300 mm^2^) and elevated area fraction (20.21%). The NTVP formulation exhibited a distinct porous architecture characterized by smaller and more well-defined pores, together with the absence of large continuous lamellar structures. This sample showed the highest pore count (n/mm^2^ = 419.5), combined with a low average void area (0.005 mm^2^) and the highest area fraction (20.44%). These results indicate that hydrogen peroxide promoted extensive structural fragmentation, limiting the development of extended lamellar domains while preserving a highly porous network, corroborating the result presented in the FTIR in the aliphatic CH region.

When observing at deeper magnification (1500×), the morphology looks more similar; however, the formulations containing tannins present more granules on the surface of the cryogels (red spots). Similar behavior has been reported for tannin-immobilized nanocellulose systems, in which tannin particles were observed coating or interacting with cellulose nanofibrils, generating discrete granular features throughout the structure [25].

### 3.4. Bulk Density

Lightweight monoliths (3 × 3 × 2.5 cm^3^), more than other materials, can be classified by their density because most of their physical properties depend on it. Therefore, three-dimensional reconstruction by photogrammetry was necessary because it enabled the precise determination of the external volume and, consequently, the density of the cryogels.

**Figure 6:**
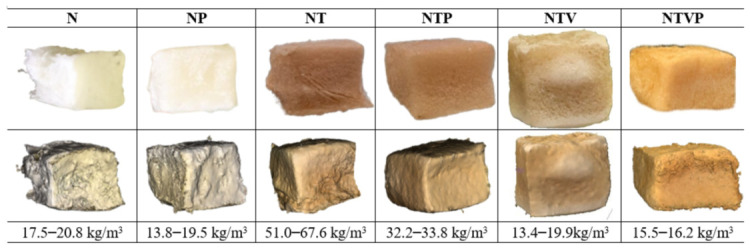
Visual appearance, reconstructed three-dimensional models of the cryogels and their respective density ranges. N, NP, NT, NTP, NTV, and NTVP correspond to the cryogel formulations described in Table 1. NT, NTP, NTV, and NTVP correspond to nanocellulose-based cryogels containing tannin, tannin + H_2_O_2_, tannin + vanillin, and tannin + vanillin + H_2_O_2_, respectively, as defined in Table 1.

In Figure 6, the visual and reconstructed images of the cryogels, as well as the densities for the cryogels, are reported. The results revealed clear density differences among the formulations, with higher values for tannin-containing systems and intermediate or lower densities for samples subjected to peroxide and/or vanillin incorporation. These density values provided a structural basis for the interpretation of the mechanical and thermal conductivity results discussed in the following sections.

The cryogels produced presented a range of values between 13.4 and 67.6 kg/m^3^ and a certain variability, which highlighted a general increase when tannin was added and a decrease when peroxide and vanillin were used. The NT cryogels showed the highest density values, indicating that tannin promoted a more compact and interconnected structure through intermolecular interactions with nanocellulose. On the other hand, NP, NTV, and NTVP showed lower density ranges, suggesting that peroxide treatment and vanillin incorporation contributed to the formation of more expanded and porous structures. The NTP cryogels presented intermediate density values, indicating that the oxidative treatment partially reduced the densification effect promoted by tannin.

### 3.5. Mechanical and Thermal Properties

The cryogels were evaluated according to their density, maximum compressive force (F_max_), elastic recovery (F_maxa_/F_maxb_), elastic modulus (MOE), and thermal conductivity, as summarized in Table 3.

The mechanical behavior of the cryogels was strongly influenced by the structural organization of the porous network, particularly by the balance between lamellar densification, fibrillar aggregation, and pore connectivity induced by tannin, vanillin, and peroxide treatments. Among the investigated systems, NT exhibited the highest density (59.99 ± 8.39 kg·m^−3^), which was directly associated with the densified morphology observed by SEM, characterized by reduced interlamellar distances and higher fiber aggregation. Consequently, this densified architecture likely increased the number of physical contact points and intermolecular interactions between fibrillar domains, resulting in higher stress transfer efficiency under compression and consequently superior Fmax and MOE values. NT also showed the highest Fmax (22.2 ± 9.38 N) and MOE (143.59 ± 57.78 kPa), confirming that tannin incorporation promotes a more compact and mechanically resistant structure. The higher standard deviation observed for NT is consistent with the heterogeneous densification typical of bio-based porous networks.

Conversely, N and NP showed the lowest density, Fmax, and MOE values, without significant statistical differences between them. These results agree with the SEM analysis, where N exhibited a lamellar porous structure, while NP presented a more fragmented morphology induced by peroxide treatment. The peroxide-treated systems presented more fragmented and less continuous architectures, as observed by SEM, suggesting that oxidative treatment partially disrupted cohesive fibrillar domains. Although peroxide promoted fibrillation and structural reorganization, the resulting network exhibited lower structural continuity and reduced load-bearing efficiency under compression.

NTP, NTV, and NTVP exhibited intermediate mechanical behavior, indicating partial structural reorganization of the cryogel network. NTP presented increased density and compressive resistance compared to N and NP, suggesting that tannin incorporation partially compensated for the structural fragmentation induced by peroxide treatment. In contrast, NTV and NTVP maintained relatively low densities while still exhibiting superior compressive performance compared to N and NP, indicating that intermolecular organization and interfacial stabilization partially compensated for the absence of network densification. This behavior is consistent with the FTIR results, which revealed modifications in the aromatic (~1510 cm^−1^) and aliphatic (~2900 cm^−1^) regions, suggesting changes in the supramolecular organization of the fibrillar network rather than the formation of well-defined covalent interaction structures. In this context, vanillin likely contributed predominantly through hydrogen bonding and aromatic interactions, promoting local structural stabilization within the porous architecture.

Despite the structural differences among formulations, all cryogels exhibited high elastic recovery (~91–96%), indicating that the porous architectures maintained substantial structural resilience after cyclic compression. This behavior suggests that the freeze-dried fibrillar networks preserved elastic deformation capability even in systems presenting partial densification or oxidative fragmentation. Thermal conductivity values ranged from 38.93 to 43.79 mW/(m·K) and were influenced not only by density but also by pore organization, fibrillar connectivity, and structural homogeneity within the cryogel network. In highly porous lignocellulosic materials, heat transport occurs through a combination of solid conduction along interconnected fibrillar domains and gaseous conduction through air-filled pores. Therefore, both porosity and internal architecture play important roles in determining thermal transport behavior. NP exhibited the lowest thermal conductivity value, which is consistent with its highly fragmented and porous morphology observed by SEM. The oxidative treatment promoted disruption of continuous fibrillar domains, limiting heat transfer pathways through the solid phase and consequently reducing thermal conduction efficiency.

Conversely, NT exhibited higher thermal conductivity due to its densified and more interconnected structure. The increased fiber aggregation and reduced interlamellar distances likely facilitated heat transfer through the solid network. However, SEM analysis also revealed a high number of smaller pores, which may have enhanced air confinement within the porous structure and reduced heat transfer through the gaseous phase. As a result, the increase in thermal conductivity was less pronounced than expected based solely on density, indicating that heat transport was influenced by both the solid network and the porous architecture. As reported in the literature, while densified fiber networks tend to enhance solid-phase heat transfer, smaller pores can reduce heat transfer through the gaseous phase by restricting air movement, thus affecting the overall thermal transport of the system [59].

Interestingly, NTVP exhibited relatively high thermal conductivity despite its low density, suggesting that thermal transport was not governed exclusively by density effects. The peroxide-assisted structural reorganization may have promoted improved pore connectivity and more homogeneous conductive pathways within the fibrillar architecture, favoring heat transfer even in a lightweight porous structure. Overall, the results indicate that thermal conductivity in nanocellulose-based cryogels is strongly dependent on the balance between porosity, structural continuity, and fibrillar organization, rather than solely on bulk density.

### 3.6. Leaching

Leaching experiments were performed to evaluate the effectiveness of tannin interaction within the nanocellulose cryogel network and to indirectly assess the structural stabilization promoted by vanillin and peroxide treatments. Since tannin retention is strongly dependent on intermolecular interactions, pore organization, and network continuity, the leaching behavior provides important insights into the stabilization mechanisms occurring within the porous architecture. Due to the relatively low concentration of phenolic compounds released into the aqueous medium, UV–Vis spectroscopy was employed for indirect quantification of tannin/vanillin leaching.

As a first step, four calibration curves used for tannin/vanillin quantification were prepared, and they showed satisfactory linearity, confirming the applicability of the Beer–Lambert law within the studied concentration range, where *x* represents the concentration of leached tannin (mg/mL), and *y* corresponds to the measured absorbance. The selected wavelengths (≈280 nm) are characteristic of π–π* transitions of phenolic aromatic structures associated with tannin and vanillin. The calibration curves were selected according to the composition of each formulation without nanocellulose. This approach allowed the use of calibration models representative of the chemical environment of each formulation (Table 4 and Table 5).

The NT system exhibited ≈77% tannin leaching, indicating weak retention of tannin within the nanocellulose matrix, mainly through intermolecular secondary bonds. After the incorporation of hydrogen peroxide (NTP), tannin leaching decreased to ≈22%, suggesting that oxidative treatment promoted the formation of additional oxygen-containing functionalities within the nanocellulosic network, such as carbonyl and possibly carboxyl groups [37]. These oxidized sites may increase the number of interaction points available for tannin immobilization, strengthening intermolecular associations and consequently improving tannin retention.

For the vanillin-containing system (NTV), complete tannin leaching (≈82%) was again observed, indicating that vanillin alone was insufficient to stabilize tannin within the matrix. In contrast, the peroxide-containing ternary system (NTVP) exhibited only ≈14% tannin leaching, demonstrating a significant improvement in tannin immobilization.

This behavior suggests that the combined presence of hydrogen peroxide and vanillin promotes structural reorganization and the formation of more conjugated and oxidized aromatic structures, reducing tannin mobility and solubility. These results are consistent with the FTIR and SEM analyses, which indicated increased intermolecular interactions and morphological reorganization in peroxide-containing systems. Overall, the leaching results demonstrate that peroxide treatment plays a central role in improving tannin retention within nanocellulose cryogels, particularly when combined with vanillin-containing systems. These findings reinforce the importance of structural organization and pore architecture in controlling the mobility and stabilization of phenolic compounds in bio-based porous materials.

## 4. Conclusions

Nanocellulose-based cryogels functionalized with tannin and modified with vanillin and hydrogen peroxide were successfully produced as sustainable porous bio-based materials.

Tannin incorporation promoted densification of the fibrillar network, resulting in increased density, compressive strength, and elastic modulus. In contrast, peroxide treatment induced fragmentation and a higher number of pores, generating lighter cryogels from which the phenolics leached with more difficulty.

The leaching experiments, indeed, demonstrated that peroxide treatment played a central role in improving tannin retention within the cryogel matrix, particularly in the NTVP system (≈14%), indicating that structural organization and pore connectivity significantly influence phenolic immobilization in porous nanocellulosic networks. Hence, the role of vanillin gains importance when the system also includes hydrogen peroxide, producing cryogels with enhanced pore surface area, thermal conductivity, and especially higher leaching resistance.

## Figures and Tables

**Table 1:** Nomenclature and chemical composition of cryogels.

Samples	Cellulose (wt%)	Tannin (wt%)	Vanillin (wt%)	H_2_O_2_
N	100	0	0	−
NP	100	0	0	X
NT	96	4	0	−
NTP	96	4	0	X
NTV	95	4	1	−
NTVP	95	4	1	X

**Table 2:** Quantitative pore analysis obtained from SEM micrographs.

Sample	Number of Pores (mm^−2^)	Total Pore Area (mm^2^)	Average Pore Area (mm^2^)
N	119.75	6.276	0.013
NP	154.5	3.477	0.006
NT	202.5	3.807	0.005
NTP	410.75	5.029	0.003
NTV	183.25	7.300	0.010
NTVP	419.5	8.323	0.005

**Table 3:** Mechanical and thermal properties of nanocellulose cryogels.

Samples	Density (Kg/m^3^)	Fmax (N)	Fmaxa/Fmaxb (%)	MOE (Kpa)	Thermal Condutivity (mW/(m·K))
N	18.85 ± 1.77 ab	2.22 ± 1.98 a	95.65 ± 1.35 a	7.50 ± 6.35 a	40.81 ± 0.44 b
NP	16.21 ± 2.97 a	2.36 ± 1.76 a	95.51 ± 1.58 a	12.81 ± 4.37 a	38.93 ± 0.45 a
NT	59.99 ± 8.39 c	22.2 ± 9.38 b	91.77 ± 1.06 a	143.59 ± 57.78 b	42.89 ± 0.62 c
NTP	33.06 ± 0.82 b	8.57 ± 3.93 ab	92.40 ± 6.04 a	22.11 ± 13.90 a	40.67 ± 0.55 b
NTV	16.27 ± 4.37 a	5.49 ± 4.51 ab	90.99 ± 5.02 a	22.18 ± 11.51 a	40.49 ± 0.14 b
NTVP	15.84 ± 0.70 a	6.72 ± 3.31 ab	94.63 ± 4.17 a	16.11 ± 6.34 a	43.79 ± 0.22 c

Different letters within the same column indicate statistically significant differences according to Tukey’s test at the 1% significance level (*p* < 0.01).

**Table 4:** Calibration curves of the four tannin/vanillin solutions.

System	Wavelength (nm)	Equation (y = ax + b)	R^2^	Cryogels
T	279.6	y = 8.3532x + 0.0871	0.9809	NT
TP	278.0	y = 10.595x + 0.0037	0.9980	NTP
TV	279.4	y = 33.934x − 0.0037	0.9963	NTV
TVP	278.9	y = 26.494x + 0.3192	0.9296	NTVP

Notes: T = tannin; TP = tannin + H_2_O_2_; TV = tannin + vanillin; and TVP = tannin + vanillin + H_2_O_2_ correspond to the calibration solutions associated with the cryogel formulations N, NT, NTP, NTV, and NTVP as defined in Table 1.

**Table 5:** Concentration of leachate (mg/mL) and tannin/vanillin loss (%).

Cryogel	Concentration (μg/mL)	Relative Concentration %
N	0	0
NT	30.76 ± 2.00	76.91 ± 5.01
NTP	8.76 ± 2.40	21.90 ± 5.99
NTV	32.71 ± 3.58	81.77 ± 8.96
NTVP	5.44 ± 0.67	13.61 ± 1.68

Notes: NT, NTP, NTV, and NTVP correspond to nanocellulose-based cryogels containing tannin, tannin + H_2_O_2_, tannin + vanillin, and tannin + vanillin + H_2_O_2_, respectively, as defined in Table 1.

## Data Availability

The data presented in this study are integrally available on request by the first and the corresponding author.

## References

[1] Le W.T., Kankkunen A., Rojas O.J., Yazdani M.R. (2023). Leakage-Free Porous Cellulose-Based Phase Change Cryogels for Sound and Thermal Insulation. Sol. Energy Mater. Sol. Cells.

[2] Zou F., Cucharero J., Dong Y., Kangas P., Zhu Y., Kaskirinne J., Tewari G.C., Hänninen T., Lokki T., Li H. (2023). Maximizing Sound Absorption, Thermal Insulation, and Mechanical Strength of Anisotropic Pectin Cryogels. Chem. Eng. J..

[3] Zhao J., Zhou J., Li H., Xiao A., Zhao J., Zhou J., Li H., Xiao A. (2022). Directional Fabricating of Flexible and Compressible Cellulose Nanofibril Composite Cryogel with Excellent Thermal Insulation, Flame-Retardancy and Radiative Cooling for Efficient Thermal Management. Cellulose.

[4] Laitinen O., Liimatainen H. (2024). Gelatin-Reinforced Cellulose Nanofiber Composite Cryogels for Effective Separation of Small Particulate Matter in Air. Mater. Des..

[5] Di Luigi M., Guo Z., An L., Armstrong J.N., Zhou C., Ren S. (2022). Manufacturing Silica Aerogel and Cryogel through Ambient Pressure and Freeze Drying. RSC Adv..

[6] Li C., Zhang X., Bao C., Zhang J., Tian Y., Shen J., Feng X. (2022). Freezing-Induced Chemical Crosslinking to Fabricate Nanocellulose-Based Cryogels for Efficient Bilirubin Removal. Sep. Purif. Technol..

[7] Baraka F., Labidi J. (2024). The Emergence of Nanocellulose Aerogels in CO_2_ Adsorption. Sci. Total Environ..

[8] Agustin M.B., Lehtonen M., Kemell M., Lahtinen P., Oliaei E., Mikkonen K.S. (2023). Lignin Nanoparticle-Decorated Nanocellulose Cryogels as Adsorbents for Pharmaceutical Pollutants. J. Environ. Manag..

[9] Dhali K., Ghasemlou M., Daver F., Cass P., Adhikari B. (2021). A Review of Nanocellulose as a New Material towards Environmental Sustainability. Sci. Total Environ..

[10] Guo R., Li H., Liu K., Xu H., Wang K., Yang Z., Zhao Y., Huan S., Si C., Wang C. (2024). Processable Pickering Emulsion for Composite Cryogel with Cellulose Nanofibrils and Nanochitin. Carbohydr. Polym..

[11] Coldebella R., Gentil M., Berger C., Costa H.W.D., Pedrazzi C., Labidi J., Delucis R.A., Missio A.L. (2021). Nanofibrillated Cellulose-Based Aerogels Functionalized with Tajuva (*Maclura tinctoria*) Heartwood Extract. Polymers.

[12] Amini M., Isari A.A., Ghasemi S., Banvillet G., Rojas O.J., Kamkar M., Arjmand M. (2025). Tailoring Pore Structure in Nanocellulose Cryogels: Enhancing Thermal and Electromagnetic Interference Shielding Properties. Carbohydr. Polym..

[13] Sahiner M., Demirci S., Sahiner N. (2024). Super Porous Carboxymethyl Cellulose–Tannic Acid (TA@CMC) Cryogels with Antioxidant, Antibacterial, and α-Glucosidase Enzyme Inhibition Abilities. Polysaccharides.

[14] Sommerauer L., Thevenon M.F., Petutschnigg A., Tondi G. (2019). Effect of Hardening Parameters of Wood Preservatives Based on Tannin Copolymers. Holzforschung.

[15] Missio A.L., Mattos B.D., Ferreira D.F., Magalhães W.L.E., Bertuol D.A., Gatto D.A., Petutschnigg A., Tondi G. (2018). Nanocellulose-Tannin Films: From Trees to Sustainable Active Packaging. J. Clean. Prod..

[16] Missio A.L., Mattos B.D., de Cademartori P.H.G., Berger C., Clovis R., Magalhães W.L.E.H., Gatto D.A., Petutschnigg A., Tondi G. (2017). Impact of Tannin as Sustainable Compatibilizer for Wood-Polypropylene Composites. Polym. Compos..

[17] Effraimopoulou E., Kalmár J., Paul G., Marchese L., Ioannou D., Paraskevopoulou P., Gurikov P. (2024). Whey Protein Isolate-Based Aerogels with Improved Hydration Properties for Food Packaging Applications. ACS Appl. Nano Mater..

[18] Wang X., Wang J., Han L., Liu B., Meng X. (2024). Vanillin-Crosslinked Gelatin-Polyvinyl Alcohol Aerogels: Improved Physicochemical Properties and Antimicrobial Activity. Food Biosci..

[19] Missio A.L., Gatto D.A., Tondi G., De Pós-graduação P., Florestal E., Federal U., Maria D.S., Maria S. (2019). Exploring Tannin Extracts: Introduction to New Bio-Based Materials. Rev. Ciência Madeira Braz. J. Wood Sci..

[20] Liu Y., Wang J., Sun Z. (2024). Aromatic Biobased Polymeric Materials Using Plant Polyphenols as Sustainable Alternative Raw Materials: A Review. Polymers.

[21] Ben Abda E., Bentis A., Amaral-Labat G., Pizzi A., Lacoste C., Koubaa A., Braghiroli F.L. (2025). Bark Tannins: Extraction Methods, Characterization, and Reactivity. Ind. Crop. Prod..

[22] Arbenz A., Avérous L. (2015). Chemical Modification of Tannins to Elaborate Aromatic Biobased Macromolecular Architectures. Green Chem..

[23] Pizzi A., Belgacem M.N., Gandini A. (2008). Tannins: Major Sources, Properties and Applications. Monomers, Polymers and Composites from Renewable Resources.

[24] Rodrigues M.B.B., do Nascimento A.S., Langone F., Muraro R.A., Lopes J.P.A., Carreno N.L.V., Pieniz S., Missio A.L., Cholant C.M. (2026). Ultrasound-Assisted Purification of *Acacia mearnsii* Tannin Extract for Application as a Corrosion Inhibitor in AISI 1045 Carbon Steel. Colloids Surf. A Physicochem. Eng. Asp..

[25] Cordeiro L.A., Soares A.K., Missio A.L., Carneiro M.E.B., de Muniz G.I.B., de Cademartori P.H.G. (2023). Nanocellulose-Based Tannin-Immobilized Biosorbent for Efficient Copper Ion Removal. Int. J. Biol. Macromol..

[26] Sadegh N., Haddadi H., Sadegh F., Asfaram A. (2023). Recent Advances and Perspectives of Tannin-Based Adsorbents for Wastewater Pollutants Elimination: A Review. Environ. Nanotechnol. Monit. Manag..

[27] Zhang L., Guan Q., Jiang J., Khan M.S. (2023). Tannin Complexation with Metal Ions and Its Implication on Human Health, Environment and Industry: An Overview. Int. J. Biol. Macromol..

[28] Zhang X., Tian Y., Chen H., Liu Y., Han S., Chang M., Zhuang J., Ma Q. (2025). Study on the Adsorption Behavior of a Cellulose Nanofibril/Tannic Acid/Polyvinyl Alcohol Aerogel for Cu(II), Cd(II), and Pb(II) Heavy Metal Ions. Nanomaterials.

[29] Li M., Guo L., Mu Y., Huang X., Jin L., Xu Q., Wang Y. (2024). Gelatin Films Reinforced by Tannin-Nanocellulose Microgel with Improved Mechanical and Barrier Properties for Sustainable Active Food Packaging. Food Hydrocoll..

[30] Ma J., Huang X., Jin L., Xu Q. (2025). Effect of Dialdehyde Nanocellulose-Tannin Fillers on Antioxidant, Antibacterial, Mechanical and Barrier Properties of Chitosan Films for Cherry Tomato Preservation. Food Chem..

[31] Missio A.L., Otoni C.G., Zhao B., Beaumont M., Khakalo A., Kämäräinen T., Silva S.H.F., Mattos B.D., Rojas O.J. (2022). Nanocellulose Removes the Need for Chemical Crosslinking in Tannin-Based Rigid Foams and Enhances Their Strength and Fire Retardancy. ACS Sustain. Chem. Eng..

[32] Eckardt J., De Nato M., Colusso E., Moro L., Šket P., Giovando S., Tondi G. (2025). Glyoxal as Single Crosslinker for Mechanically Blown, Condensed and Hydrolyzable Tannin Foams. Polymers.

[33] Rodrigues M.B.B., Lunkes N., Do Nascimento A.S., Langone F., Muraro R.A., Espíndola O.S., Pieniz S., Gatto D.A. (2026). Influence of the Extraction Medium of Tannins from *Eucalyptus* Bark on the Properties of Rigid Tannin–Furfuryl Alcohol Foams. ACS Omega.

[34] Abreu A.G., Costa J.J., Santos P.F., Duarte A.J., Vieira E.S., Moreira F.T.C. (2026). Innovations in Tannin-Based Phenolic Foams: A Review of the Research. Macromol.

[35] Wu X., Yan W., Zhou Y., Luo L., Yu X., Luo L., Fan M., Du G., Zhao W. (2020). Thermal, Morphological, and Mechanical Characteristics of Sustainable Tannin Bio-Based Foams Reinforced with Wood Cellulosic Fibers. Ind. Crop. Prod..

[36] Zhou X., Li B., Xu Y., Essawy H., Wu Z., Du G. (2019). Tannin-Furanic Resin Foam Reinforced with Cellulose Nanofibers (CNF). Ind. Crop. Prod..

[37] Tofani G., Cornet I., Tavernier S. (2021). Estimation of Hydrogen Peroxide Effectivity during Bleaching Using the Kappa Number. Chem. Pap..

[38] Sapuła P., Bialik-Wąs K., Malarz K. (2023). Are Natural Compounds a Promising Alternative to Synthetic Cross-Linking Agents in the Preparation of Hydrogels?. Pharmaceutics.

[39] Narasagoudr S.S., Shanbhag Y., Chougale R.B., Baraker B.M., Masti S.P., Lobo B. (2021). Thermal Degradation Kinetics of Ethyl Vanillin Crosslinked Chitosan/Poly(vinyl alcohol) Blend Films for Food Packaging Applications. Chem. Data Collect..

[40] Westlake J.R., Laabei M., Jiang Y., Yew W.C., Smith D.L., Burrows A.D., Xie M. (2023). Vanillin Cross-Linked Chitosan Film with Controlled Release of Green Tea Polyphenols for Active Food Packaging. ACS Food Sci. Technol..

[41] Karakurt I., Ozaltin K., Vargun E., Kucerova L., Suly P., Harea E., Minařík A., Štěpánková K., Lehocky M., Humpolícek P. (2021). Controlled Release of Enrofloxacin by Vanillin-Crosslinked Chitosan-Polyvinyl Alcohol Blends. Mater. Sci. Eng. C.

[42] Czarnecka E., Nowaczyk J., Prochoń M., Masek A. (2022). Nanoarchitectonics for Biodegradable Superabsorbent Based on Carboxymethyl Starch and Chitosan Cross-Linked with Vanillin. Int. J. Mol. Sci..

[43] Jaušovec D., Vogrinčič R., Kokol V. (2015). Introduction of Aldehyde vs. Carboxylic Groups to Cellulose Nanofibers Using Laccase/TEMPO Mediated Oxidation. Carbohydr. Polym..

[44] Meng Q., Wang H., Zhang Y., Huang X., Ke Q., Kou X. (2025). Vanillin Strengthened Complex Coacervation Behavior between Gelatin and Sodium Carboxymethyl Cellulose Endowed Improved Mechanical Properties of Microcapsules. Int. J. Biol. Macromol..

[45] Xu W., Jia M., Liu J., Lu Z., Bai G., Yan X., Li Y., Wang B., Chen L. (2025). Dynamic Phosphorescence and Real-Time Temperature Visualization via Synergy of Acetalization and Hydrogen Bonding. Laser Photonics Rev..

[46] Wassgren J., Clarke B.R., Messikh M.B., Ho C.H., Crosby A.J., Tew G.N., Carter K.R. (2025). Enhancement of Mechanical Properties of Nanocellulose Xerogels Using TEMPO-Oxidized Fibers. Carbohydr. Polym..

[47] Blanco I., Antonio L., Corrales P., Osetrov K., Uspenskaya M., Sitnikova V. (2021). The Influence of Oxidant on Gelatin–Tannin Hydrogel Properties and Structure for Potential Biomedical Application. Polymers.

[48] Zanchin A., Perbellini A., Sozzi M., Marinello F., Guerrini L. (2025). Assessment of Grapevine Bunch Withering: Advances in Fruit 3D Morphology and Colour Evaluation. Biosyst. Eng..

[49] (2021). Rigid Cellular Plastics—Determination of Compression Properties.

[50] Gustavsson M., Karawacki E., Gustafsson S.E. (1994). Thermal Conductivity, Thermal Diffusivity, and Specific Heat of Thin Samples from Transient Measurements with Hot Disk Sensors. Rev. Sci. Instrum..

[51] Gustafsson S.E. (1991). Transient Plane Source Techniques for Thermal Conductivity and Thermal Diffusivity Measurements of Solid Materials. Rev. Sci. Instrum..

[52] Tondi G. (2017). Tannin-Based Copolymer Resins: Synthesis and Characterization by Solid State 13C NMR and FT-IR Spectroscopy. Polymers.

[53] Donald L.P., Lampman G.M., Kriz G.S. (2008). Introdution to Spectroscopy.

[54] Eckardt J., Neubauer J., Sepperer T., Donato S., Zanetti M., Cefarin N., Vaccari L., Lippert M., Wind M., Schnabel T. (2020). Synthesis and Characterization of High-Performing Sulfur-Free Tannin Foams. Polymers.

[55] Łojewska J., Missori M., Lubańska A., Grimaldi P., Ziȩba K., Proniewicz L.M., Congiu Castellano A. (2007). Carbonyl Groups Development on Degraded Cellulose. Correlation between Spectroscopic and Chemical Results. Appl. Phys..

[56] Rodrigues D.d.S., Schmitt P.O., Cordeiro L.A., Rodrigues M.B.B., Ribeiro A.C.R., Bosenbecker M.W., Silva S.K.S., Carreno N.L., Gatto D.A., Silva S.H.F.d. (2025). Sustainable Films Derived from *Eucalyptus* Spp. Bark: Improving Properties Through Chemical and Physical Pretreatments. Polymers.

[57] Li L., Li Z., Wei Z., Yu W., Cui Y. (2020). Effect of Tannin Addition on Chromatic Characteristics, Sensory Qualities and Antioxidant Activities of Red Wines. RSC Adv..

[58] Coppola F., Picariello L., Forino M., Moio L., Gambuti A. (2021). Comparison of Three Accelerated Oxidation Tests Applied to Red Wines with Different Chemical Composition. Molecules.

[59] Lian X., Tian L., Li Z., Zhao X. (2024). Thermal Conductivity Analysis of Natural Fiber-Derived Porous Thermal Insulation Materials. Int. J. Heat Mass Transf..

